# A novel variant in *MYBPC3* causes hypertrophic cardiomyopathy by haploinsufficiency

**DOI:** 10.1371/journal.pone.0333096

**Published:** 2025-10-24

**Authors:** Yuanyuan Zhang, Wenyan Gong, Yusheng Cong, Xingwei Zhang, Zhelan Zheng

**Affiliations:** 1 Department of Cardiovascular Ultrasonic Center, The First Affiliated Hospital, Zhejiang University School of Medicine, Hangzhou, China; 2 School of Clinical Medicine, Affiliated Hospital of Hangzhou Normal University, Hangzhou Normal University, Hangzhou, China; 3 Key Laboratory of Aging and Cancer Biology of Zhejiang Province, School of Basic Medical Sciences, Hangzhou Normal University, Hangzhou, China; Scuola Superiore Sant'Anna, ITALY

## Abstract

**Background:**

Familial hypertrophic cardiomyopathy (HCM) is the most common genetic cardiovascular disease (CVD). Related mutations contributing to hypercontractility and poor relaxation in HCM are not completely understood.

**Purpose:**

This study aimed to explore and verify a novel variant of cardiac myosin-binding protein C (cMyBP-C, encoded by *MYBPC3*) in an HCM family.

**Methods:**

Clinical information and cardiac parameters were collected in the pedigree. Genomic DNA was extracted from peripheral blood and second-generation sequencing technology was used to investigate the proband and his family members. Subsequent sequence analysis was performed with DNAMAN software. The cardiac expression levels of MYBPC3 mRNA and cMyBP-C protein were assessed using RT-qPCR and Western blot analysis, respectively.

**Results:**

Typical interventricular septal thickening was detected in all four HCM patients without left ventricular outflow tract obstruction. The c.1042_1043insCGGCA mutation in *MYBPC3* was verified in the proband and family members. In silico analysis of the mutation revealed that c.1042_1043insCGGCA led to a shift in the sequence of nucleotides, creating a premature stop codon at the new reading frame. RT-qPCR analysis of MYBPC3 mRNA revealed a marked reduction in HCM heart compared to the normal controls (P < 0.05). Consistently, Western blot analysis showed significantly reduced expression of cMyBP-C in the pedigree in comparison with the controls (P < 0.05).

**Conclusion:**

The novel c.1042_1043insCGGCA MYBPC3 mutation is a genetic basis for HCM due to c-MyBP-C haploinsufficiency.

## Introduction

Hypertrophic cardiomyopathy is an autosomal genetic disease that occurs in the absence of common triggers such as hypertension and aortic stenosis [1]. The prevalence of HCM is 1:500 in the general population [2]. Striking cardiomegaly and stunning asymmetry with disproportionate involvement of the interventricular septum have been observed in many cases of HCM [3]. Postmortem examination of the hearts of HCM victims showed myocyte disarray accompanied by significantly increased myocardial fibrosis [4].

Many patients with HCM experience adverse clinical outcomes including heart failure (HF), arrhythmias, and sudden cardiac death. The mortality of patients with HCM is approximately 3-fold higher than that of the general population of similar ages [5]. It is the most common cause of sudden cardiac death in young athletes without any preceding symptoms.

Many efforts have been made to gain an accurate and comprehensive understanding of the molecular basis and clinical course of this disease. Pathogenic variants of genes encoding sarcomere proteins are involved in hypertrophic cardiomyopathy [6]. More than 64 genes encoding thick and thin filaments have been claimed to be causative in HCM, with varying levels of supportive evidence [7].

Here we report a novel mutation in the myosin binding protein from a sporadic pedigree. An elderly male patient in his 80s had chest tightness and dyspnea before admission. We used DNA sequencing technology and bioinformatic tools to illustrate the genetic background and possible pathophysiological mechanisms. We aimed to gain evidence on the relationship between genotype and clinical manifestations, underscoring the value of genomics in elucidating the underlying mechanism of HCM.

## Materials and methods

### Ethical approval

**Fig 1:**
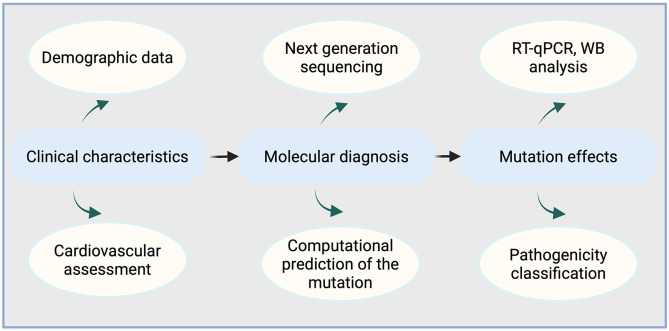
Flow diagram of the study.

The study was conducted in accordance with the Declaration of Helsinki and approved by the Ethics Committee of First Affiliated Hospital of Zhejiang University. The recruitment period started on July 15, 2022, and ended on March 15, 2023. Written consent was obtained from all participants in the study. The flowchart of the study is shown in Fig 1. Specifically, an elderly man and his adult offspring were enrolled in the study. Four patients underwent screening, and the elderly man was identified as the proband.

### Index family

Demographic data were collected for each patient on the day of admission. All patients underwent a complete cardiovascular evaluation, including cardiac physical examination, blood test for brain natriuretic peptide, 12-lead electrocardiogram (ECG), and echocardiography. The diagnostic criteria for hypertrophic cardiomyopathy were based on the recommendations of the American College of Cardiology/American Heart Association Joint Committee [8].

### Sample collection and DNA isolation

Genomic DNA from blood samples collected in ethylenediaminetetraacetic acid (EDTA) tubes was extracted using a DNeasy Blood & Tissue Kit (Cat No.69504, Qiagen, Germany) according to the manufacturer’s protocol. The concentration of genomic DNA was determined using a UV-vis/florescence spectrophotometer (ES-2, MALCOM, Japan).

### Genome sequencing

Genomic DNA extracted from peripheral blood was fragmented to an average size of ~350 bp and subjected to DNA library creation using the established Illumina paired-end protocols. The Illumina Novaseq 6000 platform (Illumina Inc., San Diego, CA, USA) was used for genomic DNA sequencing at Novogene Bioinformatics Technology Co., Ltd (Beijing, China) to generate 150-bp paired-end reads with a minimum coverage of 10× for ~99% of the genome (mean coverage of 30×). After sequencing, baseline file conversion and demultiplexing were performed using bcl2fastq software (Illumina). The resulting fastq data were submitted to in-house quality control software to remove low-quality reads, and then aligned to the reference human genome (hs37d5) using the Burrows-Wheeler Aligner (BWA) [9], and duplicate reads were marked using Sambamba tools [10]. Genomic variations were annotated using the ANNOVAR software [11].

### Sequence analysis

To study the effects of mutations on gene regulation at the genomic level, DNAMAN V8.0 software was used to carry out multiple sequence alignments.

### Western blot analysis

Cardiac tissue specimens were obtained from the proband and control subject (63-year-old male without HCM) during clinically indicated biopsy procedures. Tissues were homogenized and incubated in radioimmunoprecipitation assay (RIPA) lysis buffer. Protein aliquots (~30 μg per sample) were separated by SDS-PAGE. The blots were blocked with a non-fat dried milk solution containing 5% BSA at 37°C for 2h. The membranes were then incubated with primary antibodies against MYBPC3 (Cat No.19977-1-AP, Proteintech) and tubulin (Cat No.11224-1-AP, Proteintech) at 4°C overnight. Subsequently, the membranes were washed with TBST. HRP-binding secondary antibody was used to incubate the blots at room temperature for 2h. The Ag-Ab products were detected using the ECL system and analyzed using the ImageJ software. Two technical replicates were done for the analysis.

### Real-time reverse transcription-quantitative polymerase chain reaction (RT-qPCR)

Total mRNA was extracted using a RNeasy Mini Kit (Cat no. 74104, Qiagen) from the biopsy sample of the proband according to the manufacturer’s instructions. The quality of the RNA was evaluated via NanoDrop 260/280 ratio. mRNA (500 ng) was then converted to cDNA by reverse transcription (QuantiTect Reverse Transcription Kit, Cat no. 205313, Qiagen). cDNA (10 ng) was used for qPCR analyses using TaqMan Fast Advanced Master Mix (4444556, Applied Biosystems). The primers for RT-qPCR are listed in Supplementary Table 1. Relative expression is represented as fold change versus respective control using the cycle threshold (Ct) expressed as 2^-ΔΔCt^ relative to housekeeping gene GAPDH levels.

### Statistical analysis

Values are expressed as means ± standard deviations. Unpaired Student t-test was used to calculate the statistical significance between two groups. All the analysis was performed using GraphPad Prism version 10.4.1 (GraphPad Software Inc.; San Diego, CA, USA). Statistical significance was set to *P* < 0.05.

## Results

### Basic characteristics of the HCM family

**Fig 2:**
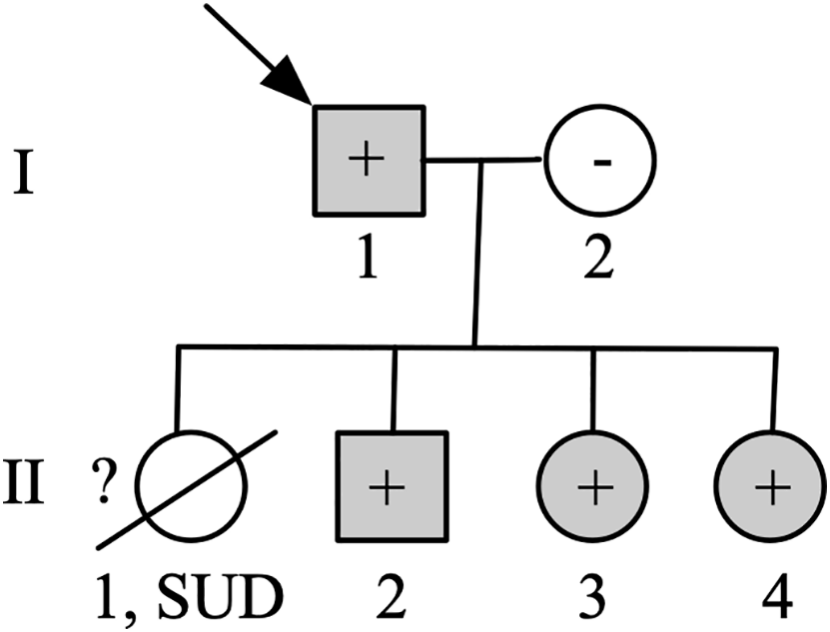
Partial pedigree chart of the HCM family. Each individual is identified with a number and generations are indicated on the left side. Patients with hypertrophic cardiomyopathy are shown in grey, unaffected individuals are shown in white, and slashes indicate a deceased relative. The index case is indicated with an arrow (I-1). The question mark indicates no genetic analysis available. Minus and plus symbols, respectively, represent genotype-negative or positive subjects. SUD means sudden unexplained death.

The partial pedigree of the family is shown in Fig 2 and the main clinical features are shown in Table 1. Briefly, the proband was diagnosed with HCM in his 50s. He had repeated episodes of heart failure and was administered diuretics. Individual II-2 was diagnosed with HCM in his 50s because of chest discomfort and palpitations. He was administered beta-blockers to relieve symptoms. Of note, individuals II-3 and II-4 remained asymptomatic thus far, without any episodes of palpitations, arrhythmia, or syncope.

**Table 1:** Baseline characteristics of the HCM patients.

Demographic characteristics	I-1	II-2	II-3	II-4
**Sex**	Male	Male	Female	Female
**Age range at diagnosis, y**	50-55	50-55	50-55	45-50
**Medical history**				
** Current smoker**	No	No	No	No
** Diabetes**	No	No	No	No
** Hypertension**	Yes	Yes	No	No
** Dyspnea or shortness of breath**	Yes	No	No	No
** Angina pectoris**	No	No	No	No
** Syncope**	No	No	No	No
** Cardiac arrest**	No	No	No	No
** Surgical myectomy**	No	No	No	No
** Heart failure episodes**	Yes	No	No	No
** NYHA class**	II	I	I	I
**Medications**				
** Beta blockers**	Yes	Yes	Yes	No
** Calcium antagonists**	No	No	No	No
** Diuretics**	Yes	No	No	No
** Warfarin**	No	No	No	No
**Biomarkers**				
** BNP (pg/ml)**	434.97	<35	<35	<35

Abbreviation: BNP = brain natriuretic peptide; HCM = hypertrophic cardiomyopathy; NYHA = New York Heart Association.

### Cardiovascular evaluation of the family

**Fig 3:**
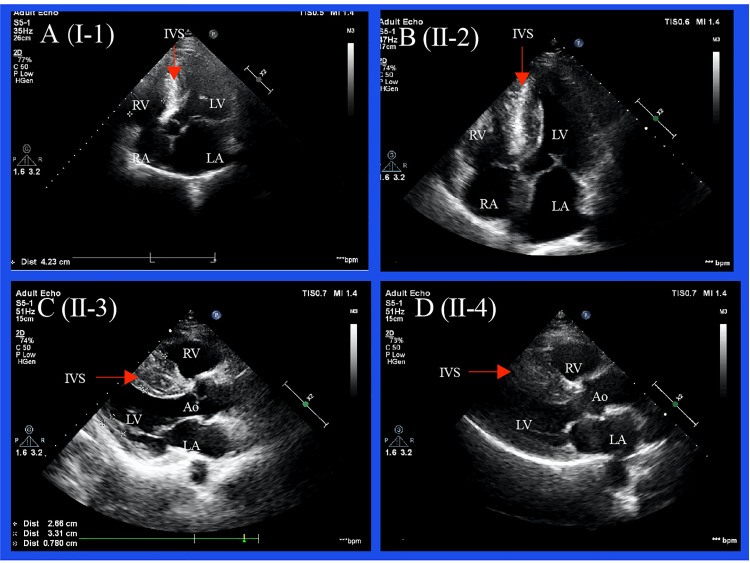
Typical echocardiography of family members. Four chamber views of I-1 and II-2. Parasternal long axis view of II-3 and II-4. LA = left atrium; LV = left ventricle; RA = right atrium; RV = right ventricle; Ao = aorta.

**Fig 4:**
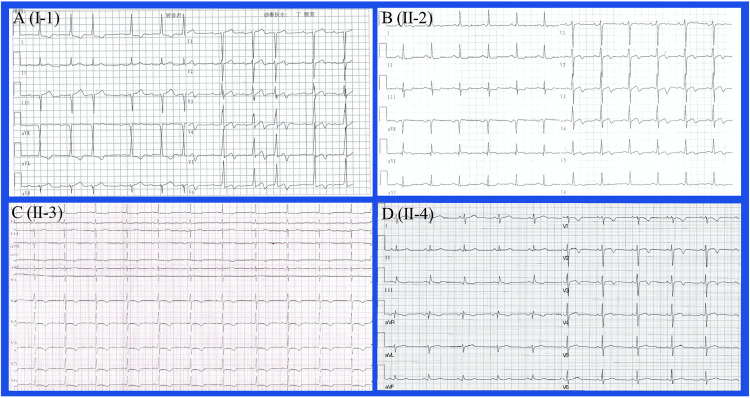
Typical ECG results show significant alterations in the hypertrophic heart. Atrial premature beat, intraventricular block, and inverted T waves in V3-6 were detected in the index case. Inverted T waves in precordial leads appeared in individuals II-2, II-3, and II-4.

Typical echocardiographic images and ECG results are shown in Figs 3 and 4, respectively. Notably, the left ventricular wall thickness increased significantly in all four patients with a maximal end-diastolic interventricular septum of ≥ 15 mm, indicating the diagnosis of HCM [8,12]. Left ventricular outflow tract obstruction was not detected in the affected members. Echocardiographic details are shown in Table 2. Electrocardiography (ECG) showed significant alterations in the hypertrophic heart. The index case shows increased QRS voltages because SV1 + RV5 and RV6 are both larger than 35 mm. Arrhythmia, including atrial premature beat and intraventricular block, occurred in the proband. T-wave inversion in at least two adjacent precordial leads was detected in individuals II-2, II-3, and II-4.

**Table 2:** Variable of transthoracic echocardiography of the pedigree.

Echocardiographic analysis	I-1	II-2	II-3	II-4
**IVSd (mm)**	39	26	26	18
**IVSs (mm)**	41	30	29	21
**LVPWd (mm)**	10	10	8	9
**LVPWs (mm)**	14	14	12	13
**IVSd/LVPWd ratio**	3.9	2.6	3.3	2.0
**EF (%)**	60	70	72	68
**FS (%)**	30	40	41	35
**LA (mm)**	44	38	33	34
**LVOT gradient (mmHg)**	9.3	7.2	6.5	5.7

Abbreviations: EF = ejection fraction; FS = fractional shortening; IVSd = interventricular septal end-diastolic thickness; IVSs = interventricular septal end-systolic thickness; LA = left atrial diameter; LVOT = left ventricular outflow tract; LVPWd = left ventricular posterior wall end-diastolic thickness; LVPWs = left ventricular posterior wall end-systolic thickness.

### Molecular diagnosis

**Fig 5:**
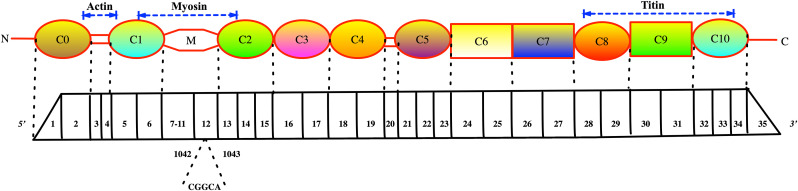
Structural representation of full-length cMyBP-C protein and *MYBPC3* exons. The studied variant is indicated in the representation. cMyBP-C is composed of 11 domains, numbered C0 to C10 from the N-terminus to C-terminus. It includes 8 immunoglobulin (IgI) domains (circles) and 3 fibronectin type III (FnIII) domains (rectangles). Four specific phosphorylation sites are located between C1 and C2, known as MyBP-C motif. The regions of interaction with other sarcomeric proteins (actin, myosin, and titin) are shown with dotted arrows. The relationship between the 35 exons of *MYBPC3* cDNA and cMyBP-C domains is indicated by dashed lines.

Mutation screening revealed a candidate mutation in *MYBPC3* in all four patients. A heterozygous mutation, c.1042_1043insCGGCA, located in exon 12, was found in the proband and his offspring (Fig 5). The base sequences of exon 12 before and after mutation are shown in the supplementary material. According to in silico analysis, the c.1042_1043insCGGCA mutation caused a shift in the reading frame beginning with proline, changing it to arginine, creating a premature stop codon at the new reading frame, and leading to an early termination of cMyBP-C at the C2 domain in Homo sapiens.

### Identification of reduced expression of cMyBP-C in HCM

**Fig 6:**
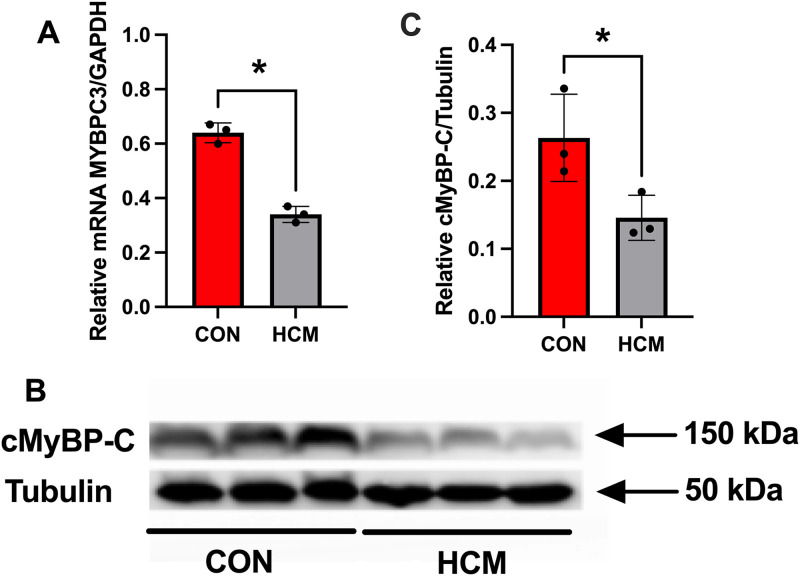
Reduced expression of MYBPC3 mRNA and cMyBP-C protein in the proband. (A) RT-qPCR analysis showing significantly decreased MYBPC3 mRNA levels in cardiac sample from the proband compared to control subjects. (B) Representative Western blot images demonstrating reduced cMyBP-C protein expression in the HCM heart relative to controls. (C) Semi-quantification of cMyBP-C protein levels normalized to tubulin. Data are presented as mean ± standard deviation (SD); ^*^P < 0.05 versus controls. HCM = hypertrophic cardiomyopathy.

RT-qPCR analysis of MYBPC3 mRNA revealed a marked reduction in the proband compared to normal controls (P < 0.05). Western blot analysis further demonstrated a significantly decreased expression of cMyBP-C protein in HCM patient relative to controls (Fig 6). According to the American College of Medical Genetics (ACMG) framework, the c.1042_1043insCGGCA in the MYBPC3 gene is interpreted as a pathogenic mutation based on the following criteria: PVS1: null variant (nonsense, frameshift, canonical ±1 or 2 splice sites, initiation codon, single or multiexon deletion) in a gene where loss of function is a known mechanism of disease; PS4: The prevalence of the variant in affected individuals is significantly increased compared to the prevalence in population controls [13].

## Discussion

In this study, we described a novel mutation in the encoding region of *MYBPC3* in an HCM family. All four patients were heterozygous carriers of the *MYBPC3* mutation here studied and exhibited thickened interventricular septum without left ventricular outflow tract obstruction. The mutation led to reduced expression of cMyBP-C in the affected heart and was supposed to be relevant to the pathogenesis of HCM. Although prior studies have reported HCM cases associated with *MYBPC3* mutation [14–16], the present study is the first to report the c.1042_1043insCGGCA mutation in exon 12.

As in the present research, the studied variant in *MYBPC3* was the main contributor to the development of HCM. Studies have shown that mutations in *MYBPC3* are relatively more benign than those with *MYH7* [17,18]. It was observed that patients with *MYBPC3* variants often have an older age of onset, along with slower progression and better prognosis [19,20]. In the current pedigree, the proband is in his 80s and thus consistent with a normal life expectancy. However, as demonstrated by the electrocardiogram, they are prone to arrhythmias such as premature atrial beat and intraventricular block. In addition, they show inverted T waves which may be signs of ischemia due to abnormal intramural microvasculature in the thickened myocardium. Therefore, periodic assessments and surveillance are required for the pedigree despite relatively mild symptoms.

Accumulating evidence indicates that pathogenic variants in genes encoding sarcomeric proteins are the main causes of HCM. Disease-causing variants in MYBPC3 account for approximately 30–40% of all those identified in HCM [21]. The protein includes 1274 amino acids and weights about 140 kDa. It comprises 11 globular immunoglobulin and fibronectin domains (C0–C10) and an extensible M-domain between C1 and C2 (Fig 5). Strikingly, most mutations are nonsense, including indels, frameshift, and splice-site mutations, leading to loss-of-function mutations [22,23]. Prior studies have shown that with the degradation of mutant transcripts by nonsense-mediated decay (NMD) and generation of premature termination codons (PTCs), the expression level of cMyBP-C is usually reduced in these patients [24]. In this study, RT-qPCR analysis revealed a marked reduction in MYBPC3 mRNA expression in the proband compared to normal controls, strongly suggesting that NMD is activated in response to the mutant transcript. Consequently, the reduction in transcript levels leads to decreased production of the encoded cMyBP-C protein, as confirmed by Western blot. This supports the concept that the mutation causes haploinsufficiency, a condition in which a single functional copy of the gene does not produce sufficient protein to maintain normal function.

Haploinsufficiency of MYBPC3 is a well-established pathogenic mechanism in HCM. The loss of adequate cMyBP-C disrupts sarcomeric integrity and contractile regulation, contributing to hypertrophic remodeling, myocyte disarray, and impaired diastolic function. While NMD protects against production of dysfunctional truncated proteins, it also reduces total protein output, tipping the balance toward disease in dosage-sensitive genes like MYBPC3.

In the present study, the proband suffered from repeated symptoms of HCM-related heart failure and was administered beta blockers and diuretics to relieve symptoms. Accumulating evidence indicates that HCM patients – even genotype-positive/phenotype-negative ones – have impaired left ventricular longitudinal strain when compared to normal controls [25]. Moreover, increased circulating cardiac biomarkers are detected in HCM patients, suggesting an increased risk of adverse cardiovascular events [26]. Additionally, endothelial dysfunction exacerbates cardiovascular disorders in HCM. Previous studies have shown interactions between the genetic backgrounds and environmental factor endothelin-1 (ET‐1) promoted the HCM phenotypes, including myocyte hypertrophy and myofibrillar disarray, both in the HCM patient-specific induced pluripotent stem cells (iPSC) and in the well‐characterized HCM mouse model–derived cardiomyocytes [27]. Another in vitro study demonstrated that gene silencing of endothelial von Willebrand Factor (vWF) prevented Ang II-induced ET-1 upregulation in aortic endothelial cells [28]. Indeed, patients with HCM present significantly raised levels of vWF [29].

According to the latest 2024 American (AHA/ACC) guidelines, cascade genetic screening is recommended among HCM family members [8]. Insights from the molecular genetic basis of HCM serve as a strong prognostic variable in evaluating adverse clinical outcomes and direct clinical management under various clinical scenarios. Sequencing technology development contributes significantly to the improvement of screening, genetic counseling, and risk stratification of HCM, holding promise for the prevention and early detection of the disease [30]. Meanwhile, it is of great significance for the differential diagnosis of diseases with similar clinical phenotypes and it may aid in risk stratification and help to establish individualized therapeutic strategies [31]. Physicians can offer valuable genetic counseling and prenatal testing, based upon a genetic diagnosis.

## Limitations and future directions

Several limitations of the current study should be acknowledged. First, this investigation was based on a single family with a relatively small number of affected individuals, which may limit the generalizability of the findings to the broader HCM population. The absence of functional studies restricts our ability to establish a direct causal relationship between the identified MYBPC3 c.1042_1043insCGGCA mutation and the observed clinical phenotype. While decreased mRNA and protein expression levels of cMyBP-C were demonstrated, the precise downstream molecular mechanisms by which haploinsufficiency contributes to myocardial remodeling and dysfunction remain to be elucidated. Meanwhile, as Western blot analysis was conducted with technical but not biological replicates, the findings are not generalizable to biological variation and should be interpreted cautiously.

Additionally, no in vitro or in vivo functional experiments were performed to validate the pathogenicity of the variant. For example, expression of the mutant construct in cardiomyocytes or the generation of a knock-in animal model could provide more definitive evidence regarding the functional consequences of the frameshift mutation. Further studies incorporating transcriptomic and proteomic profiling could also help identify altered signaling pathways and molecular networks associated with the mutation.

Future research should expand the sample size to include larger cohorts or unrelated HCM families carrying similar mutations, which would help confirm genotype–phenotype correlations. Longitudinal studies are also needed to assess disease progression and clinical outcomes in genotype-positive/phenotype-negative individuals, offering insights into penetrance and modifiers of disease expression. Ultimately, integration of comprehensive functional assays and multi-omics approaches may advance our understanding of the underlying pathophysiology and support the development of targeted therapies for mutation-specific forms of HCM.

## Conclusions

In conclusion, we performed next-generation sequencing to identify a novel frameshift mutation mapped to *MYBPC3* c.1042_1043insCGGCA, which was thought to be associated with late-onset myocardial hypertrophy and had not been reported before. Decreased cardiac expression of cMyBP-C was observed in the pedigree, leading to HCM by a possible mechanism of haploinsufficiency. These features may provide important information for physicians in clinical management of hypertrophic cardiomyopathy. Further researches on the mutation c.1042_1043insCGGCA are needed.

## Supporting information

S1 File Supplementary experimental data. (DOCX)

S2 File SNP data of the proband, II-2, II-3, and II-4. (DOCX)

## References

[1] Lopes LR, Ho CY, Elliott PM. Genetics of hypertrophic cardiomyopathy: established and emerging implications for clinical practice. Eur Heart J. 2024;45(30):2727–34. doi: 10.1093/eurheartj/ehae421 38984491 PMC11313585

[2] McKenna WJ, Crean A, Greenway S, Tadros R, Veselka J, Woo A. Hypertrophic Cardiomyopathy: Evolution to the Present, Ongoing Challenges, and Opportunities. Can J Cardiol. 2024;40(5):738–41. doi: 10.1016/j.cjca.2024.03.005 38492736

[3] Litt MJ, Ali A, Reza N. Familial Hypertrophic Cardiomyopathy: Diagnosis and Management. Vasc Health Risk Manag. 2023;19:211–21. doi: 10.2147/VHRM.S365001 37050929 PMC10084873

[4] Grassi S, Campuzano O, Coll M, Cazzato F, Sarquella-Brugada G, Rossi R, et al. Update on the Diagnostic Pitfalls of Autopsy and Post-Mortem Genetic Testing in Cardiomyopathies. Int J Mol Sci. 2021;22(8):4124. doi: 10.3390/ijms22084124 33923560 PMC8074148

[5] Sebastian SA, Panthangi V, Singh K, Rayaroth S, Gupta A, Shantharam D, et al. Hypertrophic Cardiomyopathy: Current Treatment and Future Options. Curr Probl Cardiol. 2023;48(4):101552. doi: 10.1016/j.cpcardiol.2022.101552 36529236

[6] Topriceanu C-C, Captur G. Aberrant Myocardial Dynamics in Subclinical Hypertrophic Cardiomyopathy. Circ Cardiovasc Imaging. 2024;17(4):e016572. doi: 10.1161/CIRCIMAGING.124.016572 38563165

[7] Melas M, Beltsios ET, Adamou A, Koumarelas K, McBride KL. Molecular Diagnosis of Hypertrophic Cardiomyopathy (HCM): In the Heart of Cardiac Disease. J Clin Med. 2022;12(1):225. doi: 10.3390/jcm12010225 36615026 PMC9821215

[8] Writing Committee Members, Ommen SR, Ho CY, Asif IM, Balaji S, Burke MA, et al. 2024 AHA/ACC/AMSSM/HRS/PACES/SCMR Guideline for the Management of Hypertrophic Cardiomyopathy: A Report of the American Heart Association/American College of Cardiology Joint Committee on Clinical Practice Guidelines. J Am Coll Cardiol. 2024;83(23):2324–405. doi: 10.1016/j.jacc.2024.02.014 38727647

[9] Kumar S, Agarwal S, Ranvijay. Fast and memory efficient approach for mapping NGS reads to a reference genome. J Bioinform Comput Biol. 2019;17(2):1950008. doi: 10.1142/S0219720019500082 31057068

[10] Tarasov A, Vilella AJ, Cuppen E, Nijman IJ, Prins P. Sambamba: fast processing of NGS alignment formats. Bioinformatics. 2015;31(12):2032–4. doi: 10.1093/bioinformatics/btv098 25697820 PMC4765878

[11] Wang K, Li M, Hakonarson H. ANNOVAR: functional annotation of genetic variants from high-throughput sequencing data. Nucleic Acids Res. 2010;38(16):e164. doi: 10.1093/nar/gkq603 20601685 PMC2938201

[12] Coylewright M, Cibotti-Sun M, Moore MM. 2024 Hypertrophic Cardiomyopathy Guideline-at-a-Glance. J Am Coll Cardiol. 2024;83(23):2406–10. doi: 10.1016/j.jacc.2024.04.002 38727648

[13] Miller DT, Lee K, Abul-Husn NS, Amendola LM, Brothers K, Chung WK, et al. ACMG SF v3.1 list for reporting of secondary findings in clinical exome and genome sequencing: A policy statement of the American College of Medical Genetics and Genomics (ACMG). Genet Med. 2022;24(7):1407–14. doi: 10.1016/j.gim.2022.04.006 35802134

[14] Marian AJ. Molecular Genetic Basis of Hypertrophic Cardiomyopathy. Circ Res. 2021;128(10):1533–53. doi: 10.1161/CIRCRESAHA.121.318346 33983830 PMC8127615

[15] Melendo-Viu M, Salguero-Bodes R, Valverde-Gómez M, Larrañaga-Moreira JM, Barriales R, Díez-Lopez C, et al. Hypertrophic cardiomyopathy due to truncating variants in myosin binding protein C: a Spanish cohort. Open Heart. 2024;11(2):e002891. doi: 10.1136/openhrt-2024-002891 39581692 PMC11590831

[16] Zhou N, Weng H, Zhao W, Tang L, Ge Z, Tian F, et al. Gene-echocardiography: refining genotype-phenotype correlations in hypertrophic cardiomyopathy. Eur Heart J Cardiovasc Imaging. 2023;25(1):127–35. doi: 10.1093/ehjci/jead200 37561025

[17] Abbas MT, Baba Ali N, Farina JM, Mahmoud AK, Pereyra M, Scalia IG, et al. Role of Genetics in Diagnosis and Management of Hypertrophic Cardiomyopathy: A Glimpse into the Future. Biomedicines. 2024;12(3):682. doi: 10.3390/biomedicines12030682 38540296 PMC10968563

[18] Guo X, Huang M, Song C, Nie C, Zheng X, Zhou Z, et al. MYH7 mutation is associated with mitral valve leaflet elongation in patients with obstructive hypertrophic cardiomyopathy. Heliyon. 2024;10(14):e34727. doi: 10.1016/j.heliyon.2024.e34727 39130421 PMC11315070

[19] Lehman SJ, Crocini C, Leinwand LA. Targeting the sarcomere in inherited cardiomyopathies. Nat Rev Cardiol. 2022;19(6):353–63. doi: 10.1038/s41569-022-00682-0 35304599 PMC9119933

[20] Braunwald E. Hypertrophic Cardiomyopathy: A Brief Overview. Am J Cardiol. 2024;212S:S1–3. doi: 10.1016/j.amjcard.2023.10.075 38368032

[21] Carrier L. Targeting the population for gene therapy with MYBPC3. J Mol Cell Cardiol. 2021;150:101–8. doi: 10.1016/j.yjmcc.2020.10.003 33049255

[22] Chiswell K, Zaininger L, Semsarian C. Evolution of genetic testing and gene therapy in hypertrophic cardiomyopathy. Prog Cardiovasc Dis. 2023;80:38–45. doi: 10.1016/j.pcad.2023.04.009 37137376

[23] Suay-Corredera C, Pricolo MR, Herrero-Galán E, Velázquez-Carreras D, Sánchez-Ortiz D, García-Giustiniani D, et al. Protein haploinsufficiency drivers identify MYBPC3 variants that cause hypertrophic cardiomyopathy. J Biol Chem. 2021;297(1):100854. doi: 10.1016/j.jbc.2021.100854 34097875 PMC8260873

[24] Gartzonikas IK, Naka KK, Anastasakis A. Current and emerging perspectives on pathophysiology, diagnosis, and management of hypertrophic cardiomyopathy. Hellenic J Cardiol. 2023;70:65–74. doi: 10.1016/j.hjc.2022.11.002 36403865

[25] Cavus E, Muellerleile K, Schellert S, Schneider J, Tahir E, Chevalier C, et al. CMR feature tracking strain patterns and their association with circulating cardiac biomarkers in patients with hypertrophic cardiomyopathy. Clin Res Cardiol. 2021;110(11):1757–69. doi: 10.1007/s00392-021-01848-5 33779809 PMC8563550

[26] Dungu JN, Hardy-Wallace A, Dimarco AD, Savage HO. Hypertrophic Cardiomyopathy. Curr Heart Fail Rep. 2024;21(4):428–38. doi: 10.1007/s11897-024-00654-0 38488965

[27] Tanaka A, Yuasa S, Mearini G, Egashira T, Seki T, Kodaira M, et al. Endothelin-1 induces myofibrillar disarray and contractile vector variability in hypertrophic cardiomyopathy-induced pluripotent stem cell-derived cardiomyocytes. J Am Heart Assoc. 2014;3(6):e001263. doi: 10.1161/JAHA.114.001263 25389285 PMC4338713

[28] Dushpanova A, Agostini S, Ciofini E, Cabiati M, Casieri V, Matteucci M, et al. Gene silencing of endothelial von Willebrand Factor attenuates angiotensin II-induced endothelin-1 expression in porcine aortic endothelial cells. Sci Rep. 2016;6:30048. doi: 10.1038/srep30048 27443965 PMC4957110

[29] Cambronero F, Vilchez JA, García-Honrubia A, Ruiz-Espejo F, Moreno V, Hernández-Romero D, et al. Plasma levels of von Willebrand factor are increased in patients with hypertrophic cardiomyopathy. Thromb Res. 2010;126(1):e46-50. doi: 10.1016/j.thromres.2010.01.010 20156645

[30] Ommen SR, Semsarian C. Hypertrophic cardiomyopathy: a practical approach to guideline directed management. Lancet. 2021;398(10316):2102–8. doi: 10.1016/S0140-6736(21)01205-8 34600606

[31] Miller EM, Brown E, Christian S, Kelly MA, Knight LM, Saberi S, et al. Genetic testing and counseling for hypertrophic cardiomyopathy: An evidence-based practice resource of the National Society of Genetic Counselors. J Genet Couns. 2024. doi: 10.1002/jgc4.1993 39484862 PMC12041840

