# Supplementary material for: A novel variant in MYBPC3 causes hypertrophic cardiomyopathy by haploinsufficiency
Source: PLoS One. 2025 Oct 24;20(10):e0333096. doi: 10.1371/journal.pone.0333096 (PMC12551867; doi:10.1371/journal.pone.0333096)
Supplement: S1 File — (DOCX) [file pone.0333096.s001.docx]

Supplementary materials

1. Base sequence of exon 12

Before mutation :

ggactcgaa gctggaggca ccagcagagg aggacgtgtg ggagatccta cggcaggcac ccccatctga gtacgagcgc atcgccttcc agtacggcgt cactgacctg cgcggcatgc taaagaggct caagggcatg aggcgcgatg agaagaagag cacag

After mutation:

ggactcgaa gctggaggca ccagcagagg aggacgtgtg ggagatccta cggcaggcac cCGGCAcccatctga gtacgagcgc atcgccttcc agtacggcgt cactgacctg cgcggcatgc taaagaggct caagggcatg aggcgcgatg agaagaagag cacag

2. Supplementary Table1: List of Quantitative Real-time PCR (qRT-PCR) primers

| Primer Name | Forward Sequence | Reverse Sequence | Vender or Source |
| --- | --- | --- | --- |
| cMyBP-C | 5′- AAGGGAAGGGAGAGAGGAGA -3′ | 5′- TTCCTCCACTCTGTCCTTCC -3′ | GeneParma |
| GAPDH | 5′- GAAGGTGAAGGTCGGAGT -3′ | 5′- GAAGATGGTGATGGGATTTC -3′ | GeneParma |

3. Original blot images
