# Supplementary material for: A novel variant in MYBPC3 causes hypertrophic cardiomyopathy by haploinsufficiency
Source: PLoS One. 2025 Oct 24;20(10):e0333096. doi: 10.1371/journal.pone.0333096 (PMC12551867; doi:10.1371/journal.pone.0333096)
Supplement: S2 File — (DOCX) [file pone.0333096.s002.docx]

**S2 File.** **SNP data of the proband, II-2, II-3, and II-4.**

Please refer to <https://figshare.com/articles/dataset/SNPs_of_I-1_II-2_II-3_and_II-4/29664620>
